# Scientific Items

**Published:** 1881-10-20

**Authors:** 


### SCIENTIFIC ITEMS.

**New Electrical Devices in Fire-Engine Houses.**—A Western paper says that the engine-house of No. 9, Cincinnati, is fitted up so as to show in a most remarkable manner the use of electricity. In the sleeping-room the beds, instead of being ranged against the wall, are placed in the same relation to each other as the spokes of a wheel. Running through the centre of each collection of coverlets, and attached to the under one, is a brass fastening, and from this leads a stout white cord, that, with the others joining in a common centre, forms a rope that passes up through the ceiling, where the other end, riding over a grooved wheel, is made fast to an eighty-pound weight. At the initial movement of the "little joker," that begins its round while the alarm is being sent in from the box to the tower, a catch releases the weight, and at the same instant the bed-clothing is dangling in a united bunch half-way to the ceiling. The time used, therefore, by the men in disengaging themselves from the coverlets is saved, and the amount, while in itself, when added to the other second saving devices, becomes an important factor. The service rendered by the "little joker" seems almost incredible. Operated by electricity, it performs the following wonderful feats, all of which are done simultaneously: Registering the number of the box from which the alarm is coming, and before it is sounded on the bells; it swings a bracket under the engine-boiler, and, turning on the gas, sends a half-inch-in-diameter jet a foot high through the well-seasoned kindling; the stable doors are thrown open, and at the same time a revolving wooden bar at the rear of each stall, and to which are affixed rawhides, turns rapidly, giving the horses an incentive to vacate as quickly as possible. Meanwhile, the trap-doors, thrown back by the same means, make clear the descent on slippery poles to the firemen, from whom the coverlets have been snatched. Similar electrical contrivances are in use in the Cambridge engine-houses in this State.—*Boston Journal of Chemistry.*

**Balloons in Submarine Work.**—That the balloon should be of any service in submarine operations might strike one at first as a thoroughly Hibernian idea; but, according to foreign journals, an experiment lately made in the port ot Kiel proves that heavy weights may be readily lifted from the bottom of the sea by this novel means. The balloon is made of canvas and metal plates, with an attached cistern containing carbonic acid gas compressed to a liquid state. When made fast to a sunken object, the communication between the cistern and the balloon is opened; inflation takes place; the sunken vessel, or whatever else it may be, is lifted, and can be towed away at pleasure. In the experiment at Kiel, an anchor-stone weighing 15 tons was thus lifted from a depth of 32 feet. The lifting power of the balloon, 10 feet in diameter, is said to be more than one hundred tons.—*Boston Journal of Chemistry.*

**How Long Should We Sleep?**—The vital processes of man, like those of all his fellow-creatures, are partly controlled by automatic tendencies. Some functions of our internal economy are too important to be trusted to the caprices of human volition; breathing, eating, drinking, and even love, are only semi-voluntary actions; and during a period varying from one-fourth to two fifths of each solar day the conscious activity of the senses undergoes a complete suspense; the cerebral workshop is closed for repairs, and the abused or exhausted body commits its organism into the healing hands of Nature. Under favorable conditions eight hours of undisturbed sleep would almost suffice to counteract the physiological mischief of the sixteen waking hours. During sleep the organ of consciousness is at rest, and the energies of the system seem to be concentrated on the function of nutrition and the renewal of the vital energy in general; sleep promotes digestion, repairs the waste of the muscular tissues, favors the process of cutaneous excretion, and renews the vigor of the mental faculties. —*Popular Science Monthly.*

**The Painlessness of Death.**—At birth the babe undergoes an ordeal that, were he conscious, would be more trying than a most painful death; yet he feels it not. Born in an unconscious state, the brain incapable of receiving conscious impressions, his entrance into this hitherto unknown world, is accomplished during a state of oblivion, known as Nature's anæsthesia:

> "Painless we come, whence we know not—
> Painless we go, whither we know not!"

From the earliest period of human history, death has been considered as necessarily accompanied by pain; so general is this belief, that the terms "death-agony," "last struggle," "pangs of death," etc., have been in almost universal use in every age and under all conditions of society.

Nothing could be more erroneous; the truth is, pain and death seldom go together—we mean the last moments of life. Of course, death may be preceded by weeks or even months of extreme suffering, as occurs during certain incurable diseases.—*Ibid.*

**Rendering Cotton Goods Uninflamable.**—Dr. Kedzie, of the State Board of Health of Michigan, in a recent address before a sanitary meeting in that State, made the very excellent suggestion that cotton fabrics—with special reference to articles of clothing—could be prevented from taking fire by the simple expedient of adding a little borax to the starch with which the goods in question are dressed. The quantity recommended is a teaspoonful of borax to each pint of starch after the latter is dissolved in water. The use of borax is entirely unobjectionable, being quite harmless and very cheap. The speaker showed by experiment that muslin, and even the most gauzy and inflammable textures, when treated with the borax starch, could not be made to take fire and burn with a blaze; and he most properly inferred that if cotton dresses and underclothing of women and children were prepared by this simple method, many distressing accidents and frequent loss of life from the accidental ignition of clothing might be prevented.—*Drug. Cir. and Chem. Gaz.*

